# SPATA2 and CYLD inhibit T cell infiltration into colorectal cancer *via* regulation of IFN-γ/STAT1 axis

**DOI:** 10.3389/fonc.2022.1016307

**Published:** 2022-12-02

**Authors:** Tze Guan Tan, Yulia Zybina, Cooper McKenna, Aleksandra Olow, Subhadra Jayaraman Rukmini, Michael Thomas Wong, Svetlana Sadekova, Alissa Chackerian, David Bauché

**Affiliations:** ^1^ Merck Sharpe & Dohme (MSD), Merck Research Laboratories (MRL), Singapore, Singapore; ^2^ Merck & Co., Inc., Merck Research Laboratories (MRL), South San Francisco, CA, United States

**Keywords:** colon cancer, SPATA2, CYLD, STAT1, chemokines

## Abstract

**Introduction:**

Colorectal cancer (CRC) is largely refractory to currently available immunotherapies such as blockade of programmed cell death protein-1 (PD-1).

**Results:**

In this study, we identified SPATA2 and its protein partner CYLD as novel regulators of CXC-ligand 10 (CXCL10), a T-cell-attractant chemokine, in CRC. By specifically deleting SPATA2 and CYLD in human and mouse CRC cell lines, we showed that these two proteins inhibit STAT1 accumulation and activation and subsequently CXCL10 expression in tumor cells. At steady-state, STAT1 is highly ubiquitinated in a SPATA2/CYLD-dependent manner. Finally, we demonstrated that tumor-specific deletion of SPATA2 and CYLD enhances anti-PD-1 response *in vivo*.

**Discussion:**

Our data suggest that SPATA2 and CYLD represent two potential novel targets for treatment of immune-excluded, PD-1-resistant tumors.

## Introduction

Therapies blocking programmed cell death protein-1 (PD-1) and programmed cell death ligand-1 (PD-L1) have demonstrated considerable success in treating a variety of advanced cancers (1, 2). However, a substantial number of patients manifest resistance to anti-PD-(L)1 treatment. Infiltration of tumors with T cells has been associated with favorable responses to PD-(L)1 blockade (3–6), suggesting that strategies which mitigate T cell exclusion from the tumor microenvironment (TME) might improve the efficacy of anti-PD-(L)1 therapies. Several determinants of intratumoral T cell infiltration have been described, of which tumor-intrinsic gene programs represent a crucial mechanism underlying T cell exclusion (5, 7, 8). For instance, elevated Wnt/β-catenin signaling is associated with a non-T-cell-inflamed TME across the majority of tumor types in the The Cancer Genome Atlas (TCGA) (9). Tumor-intrinsic T-cell-exclusionary pathways typically converge on a repression of type I and/or II interferons (IFNs), and thence a paucity of IFN-induced T cell chemokines such as CXC-chemokine ligand 9 (CXCL9), CXCL10, and CXCL11 in the TME (7). CXCL9/10/11 are ligands for CXC-chemokine receptor 3 (CXCR3), which is highly expressed on cytotoxic CD8^+^ T cells and IFN-γ-producing CD4^+^ T helper 1 cells, both of which provoke tumor cell killing and enhance inflammation in the TME. Thus, the CXCL9/10/11-CXCR3 axis promotes the recruitment of anti-tumor T cells into the tumor. Accordingly, targeting T-cell-exclusion pathways in malignant cells induces T cell chemokines, increases intratumoral T cell accumulation, and potentiates tumor responses to PD-(L)1 blockade (10–13).

Colorectal cancer (CRC) is the third most common cancer type in the United States and is often refractory to PD-(L)1 blockade and other immunotherapies, with only tumors harboring a defective mismatch repair system (~15% of all cases) manifesting favorable responses to anti-PD-(L)1 (14, 15).The aforementioned tumors are typically hypermutated, enriched for microsatellite instability (MSI), and display extensive T cell infiltration, while the remaining microsatellite stable (MSS) CRC tumors are often depleted of T cells (16, 17). Of note, the extent of T cell accrual within CRC tumors, regardless of MSI status, is prognostic for survival (17), suggesting that T cells are functional in CRC and stimulating T cell recruitment into the TME might sensitize MSS patients to PD-(L)1 blockade. Indeed, buttressing intratumoral T cell accumulation in murine CRC models *via* various approaches promotes tumor regression (18, 19) and overcomes resistance to anti-PD-(L)1 (20).

Here we sought to identify oncogenic pathways that mediate T cell exclusion and immune evasion in CRC. We identified SPATA2 and its interacting partner, CYLD, as tumor-intrinsic regulators of T cell chemokines, including CXCL10, *via* a novel mechanism. Genetic ablation of SPATA2 or CYLD in murine CRC tumors increased CXCL10 expression and T cell accumulation in the TME, concomitant with retarded tumor growth.

## Materials and methods

### Mice

WT Balb/cAnNTac and C.129S6(B6)-*Rag2^tm1Fwa^* N12 mice were obtained from Taconic. Mice maintained under specific pathogen-free conditions and kept in microisolators with filtered air at the research laboratories of Merck & Co., Inc., South San Francisco, CA, USA (MRL) animal facilities.

### TCGA, GTEx, and CCLE analyses

TCGA RSEM normalized gene expression data and clinical data for colorectal adenocarcinomia (COAD) and rectal adenocarcinoma (READ) were downloaded from https://tcga.xenahubs.net. A combined CXCL10/11 score was calculated for each sample using default settings in GSVA (21) on log_2_(*x* + 1)-transformed data. GEP scores were calculated as a weighted sum of expression values for the 18 constituent genes (6) on log_10_(*x* + 1)-transformed data. The Spearman correlation of each gene with CXCL9/10/11 and GEP scores was performed and *p* values corrected for multiple testing using the Benjamini-Hochberg method. CCLE RSEM TPM gene expression data were downloaded from DepMap portal (https://depmap.org/portal/) (22). Genes were binned into tertiles based on their mean expression in CCLE CRC cell lines. Genes with a correlation coefficient of < -0.15 and false discovery rate of < 0.01 with both CXCL9/10/11 and GEP and which expression in CRC cell lines fell within the highest tertile were nominated as candidates for empirical validation. Genomic alterations for *SPATA2* in TCGA CRC tumors were visualized using cBioportal (http://www.cbioportal.org/). Estimation of T cell infiltrates as a function of *SPATA2* copy number in TCGA CRC tumors was performed using TIMER (https://cistrome.shinyapps.io/timer/) (23).

For comparison of *SPATA2* expression between CRC tumors and healthy colons, TCGA and GTEx RSEM TPM gene expression and MSI status were obtained from the UCSC Xena portal ((n=19,131) UCSC Toil RNA-seq Recompute, link). TCGA tumors represent a cohort of 290 colon adenocarcinomas and GTEx 308 normal transverse (167) and sigmoid (141) colon tissue samples. TPM expression is presented on log_2_(TPM + 1) scale. *SPATA2*-high samples were defined as those with *SPATA2* expression > 10 TPM.

### Cell lines

Human colon cancer cell lines LS411N and SW480 and mouse colon cancer CT26 cell line were purchased from the American Type Tissue Collection (ATCC). LS411N was cultured in DMEM media (ThermoFisher) containing 10% heat-inactivated fetal calf serum (FCS) (GE Life Sciences), 100 U/ml penicillin (ThermoFisher), and 100 µg/ml streptomycin (ThermoFisher), while SW480 was cultured in DMEM media (ThermoFisher) containing 10% FCS, 100 U/ml penicillin, and 100 µg/ml streptomycin. Murine cell line CT26 was cultured in DMEM containing 10% FCS (Gibco). All cells were cultured in a humidified incubator at 37°C with 5% CO_2_.

### RNAi

Cells were seeded in duplicates in transfection medium [RPMI-1640 (ThermoFisher) containing 10% FCS (GE Life Sciences)] in a 96-well plate for 16-24 h such that they reach 60-70% confluency at the time of siRNA transfection. Cells were transfected successively on days 1 and 2 post-seeding with 50 nM of TriFECTa^®^ RNAi Kit (Integrated DNA Technologies), a pool of 3 unique siRNAs, and 0.5 μl of Lipofectamine^®^ RNAiMAX Reagent (Invitrogen) per well as per manufacturer instructions. On day 3, media was replaced with RPMI-1640 (ThermoFisher) containing 0.5% (w/v) bovine serum albumin (Sigma-Aldrich) and 1 ng/ml or 10 ng/ml recombinant human IFN-γ (R&D Systems) for LS411N and SW480 respectively. Conditioned media were harvested for chemokine measurements 48 h post-IFN-γ-treatment.

### Generation of KO cell lines

The generation of KO CT26 cells was outsourced to ThermoFisher. 10^5^ CT26 cells were transfected with 1 μg of Cas9 nuclease, 200 ng of gRNA (listed below) and 10 pmol ss-Oligo with Neon^®^ transfection condition 4 according to the Neon^®^Transfection System (#MPK10025) protocol. Two days post-transfection, CT26 cells were harvested and processed according to the GeneArtTMGenomic Cleavage Detection Kit (#A24372) protocol. Single cell isolation was performed *via* FACS sorting. Clones were expanded and sequenced *via* next generation sequencing. Four complete *Spata2*-KO and three complete *Cyld*-KO clones were identified and used in this study. SPATA2 KO was confirmed by Next Generation Sequencing (NGS) and CYLD protein knockout was confirmed by Western blot.

CYLD.1 CCGACTAAGTAAAGGCCTCC; CYLD.2 GTACATCCAAGACCGTTCTG; CYLD.3 GTTGGCAATTACCAACTGTG

SPATA2.1TCAGCCGAAATCGATATAAA; SPATA2.2 CTATGTCAAGTCCACGTTGC SPATA2.3 CATCTGCTCACACTCGACCT

### STAT1 and NF-κB reporter cell lines

For STAT1 reporter cell lines: 2 x 10^5^ CT26 cells were transfected with polybrene at 4 μg/ml (Sigma Aldrich) and 10^6^ pGreenFire1-STAT1-EF1-Puro lentiviral particles (System Biosciences). Transfected cells were selected with puromycin at 0.4 μg/ml (Gibco).

For NF-κB reporter cell lines: 2 x 10^5^ CT26 cells were electroporated with 1 μg of pNL3.2.NF-κB-RE[NlucP/NF-κB-RE/Hygro] Vector (Promega) using 4D-Nucleofector X Unit (SE solution, program DS-120) (Lonza). Transfected cells were selected with hygromycin B at 400 μg/ml (Gibco).

5 x 10^4^ cells were incubated at 37°C for two hours in serum-free DMEM media (Gibco). Then cells were stimulated with 1 ng/ml of recombinant mouse IFN-γ (Biolegend) for 5 hours. STAT1-reporter cells and NF-κB-reporter cells were mixed with 1:1 (v/v) of Bright Glo Luciferase assay and Nano-Glo luciferase assay system respectively. Signal was measured on the SpectraMax i3 (Molecular Devices).

### Cell proliferation *in vitro*

10^4^ cells were seeded in a 96-well plate and incubated for two days in an Incucyte (Essen Bioscience). Pictures were acquired every three hours and percentage of confluency was automatically calculated using Incucyte software (Essen Bioscience).

### *In vivo* studies

For syngeneic tumor experiments, 8-12 week-old Balb/c mice were subcutaneously injected with either 10^6^ CT26 cells in 100ul on the right flank. Tumor diameter was measured using electronic calipers and tumor volume calculated using the equation 0.5 x length x width^2^, where the length was the longer dimension. Mice were randomized to treatment groups when tumors reached ~100 mm^3^. Mice were treated twice a week intraperitoneally (i.p.) with antibodies at 10 mg/kg. Clone DX400 engineered onto mouse IgG1 isotype with D265A mutation to produce anti-PD-1 was published previously (24).

### Tumor cell isolation

Subcutaneous tumors were excised, transferred into 5 ml of PBS and mechanically dissociated using gentleMACS dissociator (Miltenyi), program m_impTumor_01.

### Flow cytometry

Cells were resuspended in PBS and stained on ice for 30 minutes in the dark with a fixable viability stain (BD Bioscience). Then, cells were resuspended into the stain buffer (BSA) (BD bioscience) and stained on ice for 30 minutes with various combinations of directly fluorochrome-conjugated antibodies. Antibodies used for flow cytometry were from BD Biosciences, Biolegend, or ThermoFisher, and include CD45 APC-Cy7 (clone 30-F11), CD8a BUV737 (clone 53-6.7), TCRb PE (clone H57-597), CD4 BUV395 (clone RM4-5), CD25 BV711 (clone PC61), CD11b AF700 (clone M1/70), Ly6G PercpCy5.5 (clone 1A8), CD11c PeCy7 (clone N418), Nkp46 FITC (clone 29A1.4), F4/80 PE-CF594 (clone T45-2342) Ly6C BV421 (clone AL-21), CXCR3 APC (clone CXCR3-173), Class I MHC FITC (clone 34-1-25) and PD-L1 BV421 (clone MIH5). For all samples, acquisition was performed on Fortessa flow cytometer (BD). Data were analyzed using FlowJo software (Tree Star).

### Co-immunoprecipitation and western blot

10^6^ cells were incubated at 37C for two hours in serum-free DMEM media (Gibco). Then cells were stimulated with 1ng/ml of recombinant mouse IFN-γ (Biolegend) for 30 minutes. Cells were lysed for 5min on ice with IP lysis buffer (ThermoFisher) supplemented with protease and phosphatase inhibitor cocktail (ThermoFisher). Protein concentration was measured using Pierce BCA assay kit according to manufacturer’s protocol (ThermoScientific). STAT1 immunoprecipitation was performed using Pierce Co-Immunoprecipitation (Co-IP) kit (ThermoScientific) according to manufacturer’s protocol. Briefly, STAT1 (D1K9Y) (Cell Signaling Technology) antibody was immobilized on beads for two hours then washed several times. Beads were incubated overnight with 400ug of unstimulated protein lysate then washed. Proteins were eluted with Elution buffer prior to Western blot analysis. Protein lysates or co-IP were mixed with NuPage LDS sample buffer (4X) and NuPage sample reducing agent (10X) (Invitrogen) and heated at 95C for 5min. Proteins were loaded on 4-12% gradient SDS-PAGE gels (Invitrogen) and transferred onto nitrocellulose membranes. Membranes were blocked in 5% BSA (w/v) in PBS for 1 hour then incubated overnight with primary antibodies purchased from Cell Signaling Technology: Stat1 (D4Y6Z), phosphor-Stat1 (Tyr701) (58D6), Beta-actin (13E5), NF-κB p65 (D14E12), Phospho-NF-κB p65 (Ser536) (93H1), JAK1 (D1T6W), IκBa (44D4), Ubiquitin (P37). Membranes were washed three times 10min in PBS supplemented with Tween-20 then incubated for 1 hour with anti-rabbit HRP or anti-mouse HRP (Cell Signaling Technology) secondary antibodies. The detection was performed using SuperSignal West Pico Plus Chemiluminescent substrate (ThermoScientific). Band intensity was calculated using ImageJ software.

### Enzyme-linked immunosorbent assay

2 x 10^4^ cells were incubated with Fludarabine (Tocris Bioscience) at 37C for two hours in complete culture media. Then cells were stimulated with 1ng/ml of recombinant mouse IFN-γ (Biolegend) for 24 hours. Mouse CXCL10 protein concentration was measured from supernatants mouse IP-10 ELISA kit (Invitrogen) according to manufacturer’s protocol.

### Luminex

CXCL9, CXCL10, and CXCL11 levels in the conditioned media of RNAi-treated human cell lines were measured using the Bio-Plex Pro Human Chemokine Assays kit (Bio-Rad) and DropArray Bead Plates (Curiox) on the Bio-Plex 200 system (Bio-Rad) as per manufacturer instructions.

### Total RNA isolation from tissues and cells and subsequent RT-QPCR gene expression analysis using the Fluidigm Biomark^®^ platform

For real-time PCR analysis, total RNA was isolated by either of two methods. Organs were homogenized in RNA STAT-60 (Tel-Test Inc.) with a polytron homogenizer and then RNA extraction was performed with the MagMAX^®^-96 for Microarrays Kit (Thermo Fisher Scientific) per manufacturer’s instructions. For cellular samples, RNA was isolated using the ARCTURUS^®^ PicoPure^®^ RNA Isolation Kit per manufacturer’s instructions (Thermo Fisher Scientific).

DNase-treated total RNA was reverse-transcribed using QuantiTect Reverse Transcription (Qiagen) per manufacturer’s instructions. Primers were obtained commercially from Thermo Fisher Scientific. Gene specific pre-amplification was done on at least 2 ng cDNA per Fluidigm Biomark^®^ manufacturer’s instructions (Fluidigm). Real-time quantitative PCR was then done on the Fluidigm Biomark^®^ using two unlabeled primers at 900 nM each and 250 nM of FAM-labeled probe (Thermo Fisher Scientific) with Taqman Universal PCR Master Mix containing UNG. Samples and primers were run on either a 48.48 array or 96.96 array per manufacturer’s instructions (Fluidigm). Ubiquitin b levels were measured in a separate reaction and used to normalize the data by the ΔCt method. (Using the mean cycle threshold value for ubiquitin b and the gene of interest for each sample, the equation 1.8 ^ (Ct ubiquitin b minus Ct gene of interest) x 10^4^ was used to obtain the normalized values.). Primer references sequences are available on demand.

### Statistics

One-way ANOVA, one-sample Student’s *t* test, paired Student’s *t* test, and unpaired Student’s *t* test were used to calculate statistical significance as indicated in the respective figure legends unless otherwise stated. For RNA-seq data, Wilcoxon rank-sum test was used to compare SPATA2 expression across tissue types and Spearman correlation used to assess correlation of genes with gene signatures of interest. ns, Not Significant, * p<0.05, ** p<0.01, *** p<0.001, **** p<0.0001. Statistics were performed using GraphPad Prism 7 software unless otherwise stated.

## Results

### Identification of SPATA2 as a novel regulator of CXCL9/10/11 in CRC

**Figure 1:**
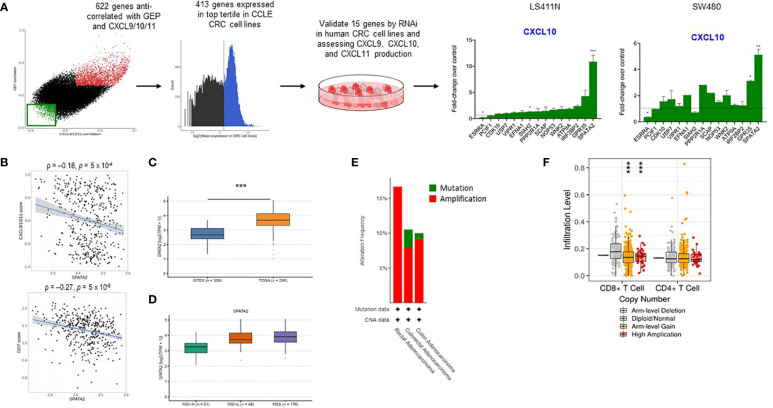
Identification of *SPATA2* as a novel regulator of CXCL10 in colon cancer. **(A)** Overview of workflow for identifying potential novel regulators of CXCL9/10/11 and T-cell-inflamed GEP. Gene expression data from TCGA CRC patients were used to identify genes that were anti-correlated with both CXCL10/11 and GEP (left panel). The aforementioned genes were filtered for high expression in CCLE CRC cell lines to enrich for tumor-intrinsic targets, and 15 candidates were selected for *in vitro* validation using RNAi and production of CXCL9, CXCL10, and CXCL11 as the readout (middle two panels). Fold-change of CXCL10 secretion elicited by knockdown of each indicated target relative to control-siRNA-treated cells for indicated cell lines are shown (right panel). Bars are ordered based on the fold-change values for CXCL10 in LS411N. Data are representative of 2-6 independent experiments. **(B)** Plots of CXCL9/10/11 (top) or T-cell-inflamed GEP (bottom) scores versus *SPATA2* expression in TCGA CRC samples. Spearman correlation coefficients (ρ) and *p* values adjusted for multiple testing by Benjamini-Hochberg method are indicated. **(C)** Transcript levels of *SPATA2* in TCGA CRC tumors versus healthy colon in GTEx. **(D)** Transcript levels of *SPATA2* in TCGA MSS CRC tumors versus MSI CRC tumors. **(E)** Frequency of the indicated genomic alterations in *SPATA2* in TCGA CRC tumors. **(F)** Relative abundance of the indicated T cell populations in TCGA CRC tumors inferred using TIMER. Mean + SEM **(B)**; Boxplots showing median and interquartile range **(E)**. * p<0.05, ** p<0.01, *** p<0.001 **(B, C, E)**; One-sample Student’s *t* test **(B)**, Wilcoxon rank-sum test **(C, D)**.

To identify tumor-intrinsic determinants of T cell recruitment into the TME, we adopted a three-step approach, integrating *in silico* analyses of gene expression data of CRC patients in TCGA and CRC cell lines in the Cancer Cell Line Encyclopedia (CCLE) (22) with *in vitro* validation of targets (**Figure 1A**, **Supplementary Table 1**). First, we identified genes which expression are anti-correlated with that of CXCL9/10/11 and T cell infiltration across CRC samples in TCGA. To quantify T cell infiltration in tumors, we scored each sample for a T-cell-inflamed gene expression profile (GEP), comprising 18 genes related to T cell effector function, IFN-γ signaling, and antigen presentation, that were previously reported to be strongly associated with responses to pembrolizumab, an antibody targeting PD-1 (5, 6). Second, we filtered the top genes anti-correlated with both CXCL9/10/11 and GEP for genes that fell into the highest tertile of expression in CCLE CRC cell lines. This step enriches for drivers of T cell exclusion that are highly expressed in tumor cells and therefore likely tumor-intrinsic. Third, we selected 15 genes from the candidate list from the second step for empirical validation. Each candidate gene was knocked down in two human CRC cell lines, LS411N and SW480, by RNA interference (RNAi), and the secretion of CXCL9, CXCL10, and CXCL11 was assessed. Knockdown of *SPATA2* elicited the most profound induction of chemokine production over the control in both cell lines in the presence of IFN-γ (**Figure 1B**, **Supplementary Figure 1**). Further analyses of *SPATA2* in TCGA genomics data revealed that *SPATA2* expression is negatively correlated with that of CXCL9/10/11 and GEP in CRC tumors (**Figure 1B**) and often elevated in tumors relative to healthy colons in Genotype-Tissue Expression (GTEx) (**Figure 1C**). Notably, *SPATA2* is over-expressed in MSS CRC compared to MSI-high (MSI-H) CRC (**Figure 1D**). In addition, genomic alterations in *SPATA2* occur in a subset of CRC patients and are largely restricted to gene amplification (~7%) (**Figure 1E**), and CRC tumors harboring increased copy numbers of *SPATA2* display reduced levels of T cell infiltration as inferred by TIMER (23) (**Figure 1F**). The collective evidence therefore suggest that *SPATA2* over-expression is associated with a non-T-cell-inflamed TME in CRC, and that SPATA2 might mediate T cell exclusion by inhibiting the production of CXCL9, CXCL10, and CXCL11 from tumor cells.

### SPATA2 and CYLD inhibit CXCL9, CXCL10 and CXCL11 expression in human and mouse cancer cell lines

**Figure 2:**
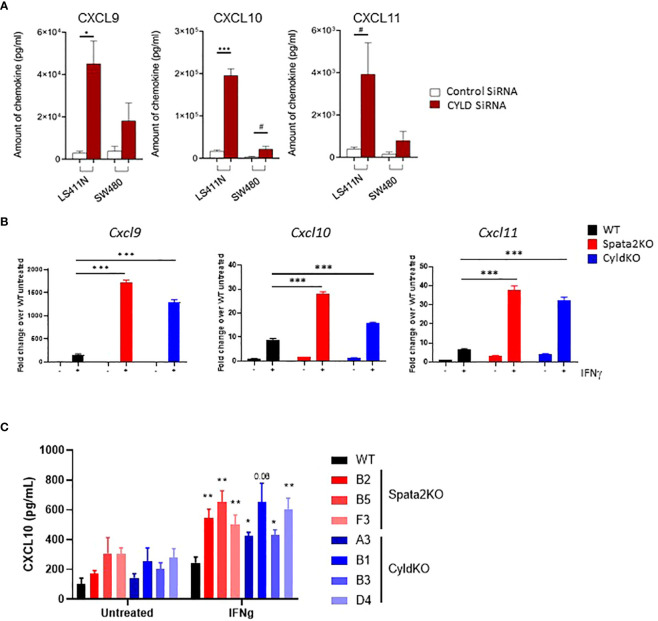
SPATA2 and CYLD inhibit CXCL9, CXCL10 and CXCL11 expression in human and mouse cancer cell lines. **(A)** Indicated human colon cancer cell lines were transfected with siRNA directed against *CYLD*. CXCL9, CXCL10 and CXCL11 protein levels were measured in the conditioned media of IFN-γ-stimulated cells by Luminex. Absolute protein levels are represented. Data are representative of 3 independent experiments. * p<0.05, *** p<0.001, ^#^ p<0.1 (Paired Student’s *t* test). **(B)** *Cxcl9*, *Cxcl10*, and *Cxcl11* gene expression were measured at the indicated time points following IFN-γ stimulation. *Spata2*-KO clone B5 and *Cyld*-KO clone D4 are represented. Results are representative of 1 out of 2 independent experiments. *** p<0.001 (Paired Student’s *t* test of IFN-γ-stimulated KO vs. WT cells). **(C)** CXCL10 protein secretion from WT vs. *Spata2*-KO and *Cyld*-KO CT26 clones. Results are representative of 1 out of 2 independent experiments. * p<0.05, ** p<0.01 (Paired Student’s *t* test vs. IFN-γ-treated WT cells). Mean ± SEM.

Initially identified as a gene involved in spermatogenesis (25), recent studies have demonstrated that SPATA2 plays a key role in regulating tumor necrosis factor (TNF) signaling (26–29). Specifically, SPATA2 recruits and activates CYLD – a deubiquitinase that attenuates TNF-induced NF-κB and mitogen-activated kinase (MAPK) signaling by removing linear and K63-linked ubiquitin chains from components of the TNF receptor signaling complex (26–29). To determine whether CYLD exerts a similar effect on tumor-intrinsic chemokine production as SPATA2, we transiently knocked down *CYLD* by RNAi and measured CXCL9, CXCL10, and CXCL11 secretion. We confirmed that downregulation of *CYLD* phenocopies *SPATA2* knockdown in strongly inducing chemokine production in response to IFN-γ (**Figure 2A**). To further validate this finding in a mouse CRC cell line, we generated *Spata2-* and *Cyld-*knockout (KO) CT26 clones and corroborated our findings in human cell lines, wherein both *Spata2*- and *Cyld*-KO CT26 lines manifested reduced *Cxcl9*, *Cxcl10* and *Cxcl11* gene expression (**Figure 2B**) and CXCL10 protein expression upon IFN-γ stimulation (**Figure 2C**). Thus, our data indicate that SPATA2 and CYLD inhibit the expression of T-cell chemokines CXCL9, CXCL10 and CXCL11.

### SPATA2 and CYLD regulate STAT1 accumulation and activation in CT26 cells

**Figure 3:**
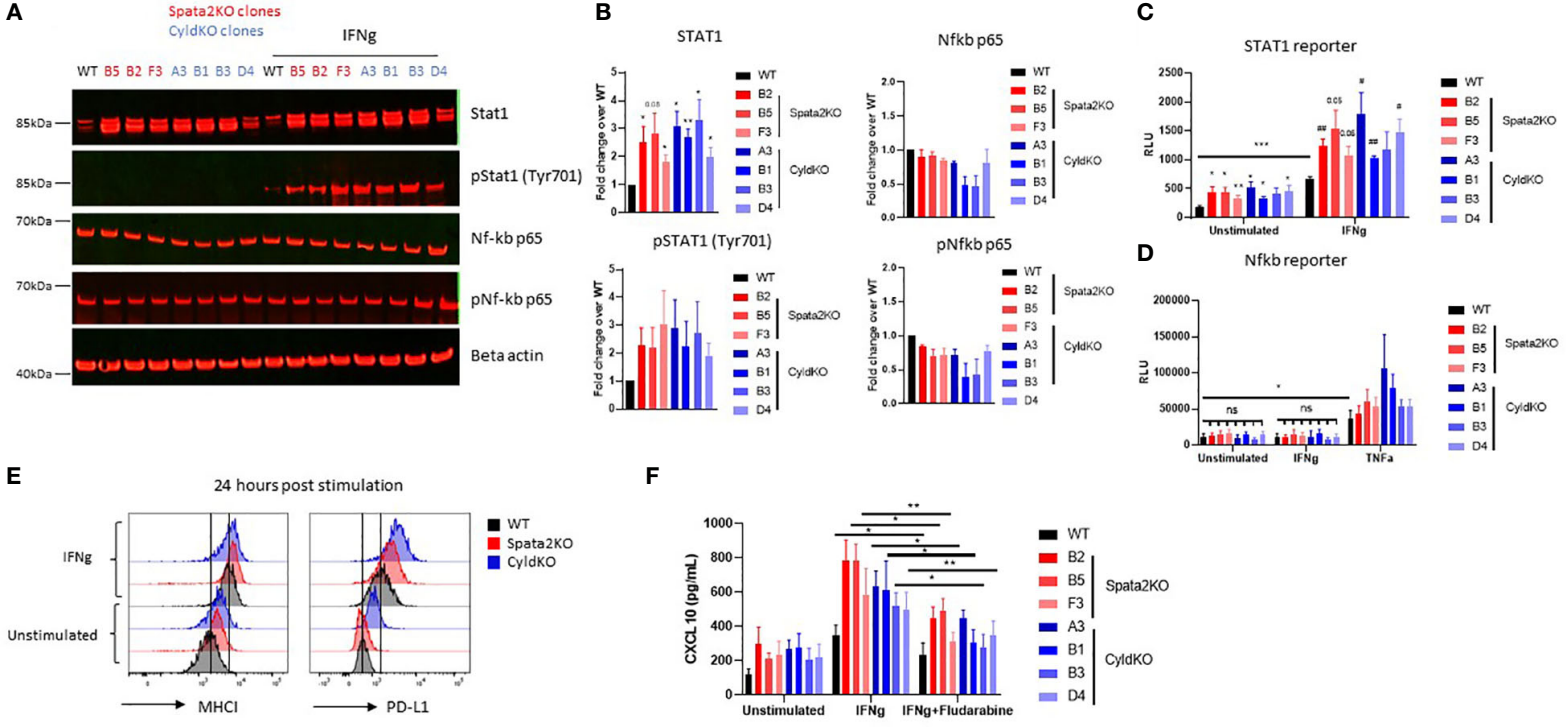
SPATA2 and CYLD regulate STAT1 accumulation and activation in CT26 cells. **(A)** STAT1, phospho-STAT1, NF-κB p65 and phospho-NF-κB p65 protein expression was measured by Western blot in untreated or IFN-γ-stimulated WT, *Spata2-*KO, and *Cyld*-KO CT26 clones. **(B)** STAT1 and NF-κB p65 protein quantification from Western blots at baseline (top panel); phospho-STAT1, and phospho NF-κB p65 protein quantification from Western blots from IFN-γ-stimulated samples (bottom panel). * p<0.05, ** p<0.01 (Paired Student’s *t* test of each condition vs. WT cells). Results are representative of 3-5 independent experiments **(A, B)**. **(C)** STAT1 activity at baseline and in response to IFN-γ was measured in CT26 WT and KO cells transfected with STAT1-reporter lentiviral particles. Results are representative of 5 independent experiments. * p<0.05, ** p<0.01, *** p<0.001 (Paired Student’s *t* test of each condition vs. unstimulated WT cells). ^#^ p<0.05, ^##^ p<0.01 (Paired Student’s *t* test of stimulated KO cells vs. IFN-γ-treated WT cells). **(D)** NF-κB activity at baseline and in response to IFN-γ or TNF-α was measured in CT26 WT and KO cells transfected with a NF-κB reporter plasmid. Results are representative of 4 independent experiments. ns: not significant, * p<0.05 (Paired Student’s *t* test). **(E)** Class I MHC and PD-L1 expression was measured by flow cytometry 24 hours after IFN-γ stimulation. Results are representative of 3 independent experiments. **(F)** CXCL10 protein levels in the supernatant of the indicated CT26 clones 24 hours after treatment with IFN-γ or IFN-γ+ Fludarabine was measured by ELISA. * p<0.05, ** p<0.01 (Paired Student’s *t* test). Mean ± SEM.

Previous reports had shown that IFN-γ induces CXCL10 production *via* activation of STAT1 and NF-κB (30). Therefore, we hypothesized that SPATA2 and CYLD are suppressing chemokine production in tumor cells by modulating NF-κB and/or STAT1 signaling. At baseline, we observed an over-expression of STAT1 but not the p65 subunit of active NF-κB (**Figures 3A, B**) in both *Spata2-*KO and *Cyld-*KO CT26 cells compared to wildtype (WT) cells. Since phosphorylation of STAT1 and p65 are required for their transcriptional activity, we assessed the levels of phosphorylated (phospho) STAT1 and p65 in *Spata2*- and *Cyld*-KO cells relative to WT cells, and found an enrichment of phospho-STAT1 but not phospho-p65 in KO cells (**Figures 3A, B**) upon IFN-γ treatment, indicative of augmented IFN-γ-STAT1 signaling in the absence of SPATA2 or CYLD. Of note, neither the expression of JAK1, a tyrosine kinase required for IFN-γ signaling, nor that of IκBα, an inhibitor of NF-κB, was regulated by SPATA2 and CYLD (**Supplementary Figure 2**). To confirm the inhibition of STAT1 activation by SPATA2 and CYLD, we generated STAT1 reporter cells *via* lentiviral transduction of STAT1-responsive transcriptional elements (SRE) driving luciferase expression. Using this more sensitive approach, we found that STAT1 is indeed over-activated and binds more to SRE in *Spata2-*KO and *Cyld-*KO CT26 cells compared to WT cells, both at baseline and in response to IFN-γ (**Figure 3C**). Similarly, we generated NF-κB reporter cells and corroborated the absence of NF-κB overactivation in cells lacking SPATA2 or CYLD, with or without IFN-γ exposure (**Figure 3D**). We noted a trend towards increased NF-κB activity in TNF-treated *Spata2-*KO and *Cyld-*KO cells, concordant with the aforementioned descriptions of SPATA2 and CYLD working in concert to limit the TNF-NF-κB pathway.

IFN-γ-inducible genes include chemokines such as CXCL9, CXCL10 and CXCL11, but also immunomodulatory surface molecules such as MHC class I and PD-L1 (18). In accordance with STAT1 upregulation, surface expression of Class I MHC and PD-L1 was elevated in *Spata2-*KO and *Cyld-*KO CT26 cells relative to WT cells in response to IFN-γ stimulation (**Figure 3E**). We therefore hypothesized that increased T-cell chemokine expression upon ablation of SPATA2 or CYLD is driven by elevated IFN-γ-STAT1 signaling in tumor cells. Consistent with this hypothesis, *Spata2-*KO and *Cyld-*KO CT26 cells incubated with Fludarabine – previously reported to inhibit STAT1 (31)– demonstrated markedly diminished levels of CXCL10 secretion upon IFN-γ stimulation (**Figure 3F**). However, Fludarabine treatment did not completely abrogate CXCL10 production, suggesting that STAT1 partially drives CXCL10 over-expression in *Spata2*-KO and *Cyld*-KO CT26 cells (**Figure 3F**). We therefore surmised that SPATA2 and CYLD suppress the production of T-cell chemokines from tumor cells, in part by regulating STAT1 protein levels and restraining STAT1 signaling (but not NF-κB signaling) emanating from IFN-γ stimulation.

### Decreased poly-ubiquitination of STAT1 in SPATA2- and CYLD-deficient CT26 cells

**Figure 4:**
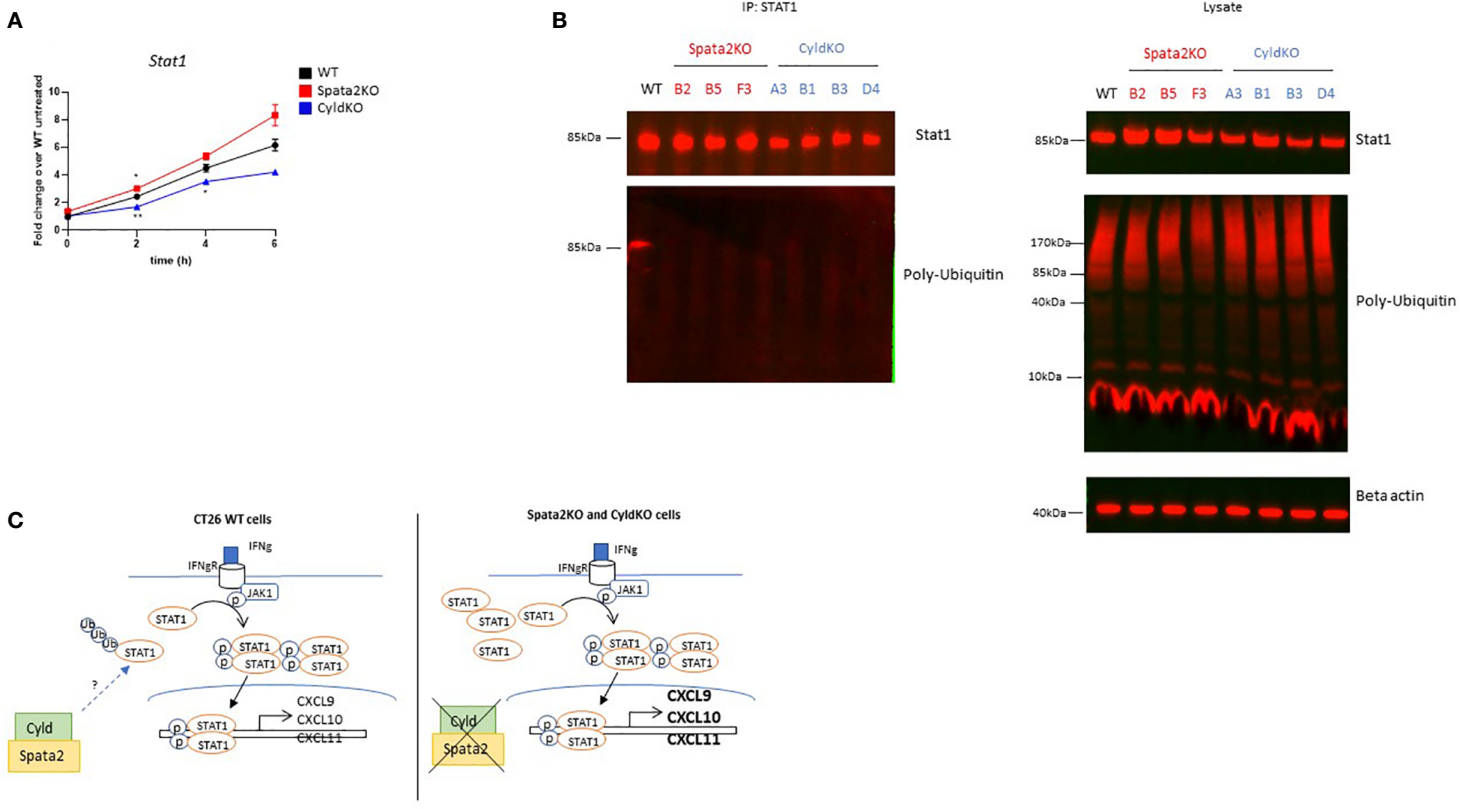
Decreased poly-ubiquitination of STAT1 in *SPATA2*- and *CYLD*-deficient CT26 cells. **(A)** Cells were stimulated with IFN-γ for 0, 2, 4 and 6 hours as indicated. *Stat1* gene expression was measured by real-time quantitative PCR (RT-qPCR). Fold change over untreated WT cells represented. Results are representative of 1 out of 2 independent experiments. Mean ± SEM. * p<0.05, ** p<0.01 (Unpaired Student’s *t* test for each timepoint) **(B)** Immunoprecipitation of STAT1 in unstimulated cells followed by Western blot of indicated protein partners (left). Input controls are shown in the right panel. Results are representative of 2 independent experiments. **(C)** Proposed mechanism of action by which SPATA2 and CYLD regulate IFN-γ-STAT1-CXCL10 axis in CRC cells.

We next sought to define the mechanisms by which SPATA2 and CYLD restrict IFN-γ-STAT1 signal transduction. To test the hypothesis that SPATA2 and CYLD regulate *Stat1* transcription, we stimulated CT26 cells with IFN-γ for 0, 2, 4 and 6 hours and measured *Stat1* gene expression (**Figure 4A**). *Stat1* mRNA levels were slightly and not significantly overexpressed in *Spata2*-KO compared to WT cells at 4 and 6 hours post-stimulation. However, the opposite trend was observed in *Cyld*-KO cells,suggesting that STAT1 protein accumulation in the absence of SPATA2 or CYLD results from post-transcriptional mechanisms. Since CYLD has deubiquitinase activity towards Met1- and K63-linked ubiquitin chains, and to a lesser extent, K48-linked ubiquitin (26–29, 32), we investigated whether STAT1 ubiquitination is affected in the absence of SPATA2 or CYLD. Unexpectedly, when we immunoprecipitated STAT1 from cell lysates, we found that STAT1 was poly-ubiquitinated in WT cells but not in *Spata2*-KO and *Cyld*-KO CT26 cells (**Figure 4B**). Notably, total levels of protein with polyubiquitination were similar across both WT and KO cell lines (**Figure 4B**). Taken together, we demonstrated that SPATA2 and CYLD attenuate STAT1 accumulation and activation in CRC cells, resulting in inhibited expression of the T-cell chemokines CXCL9, CXCL10, and CXCL11 (**Figure 4C**).

### CYLD deficiency enhances anti-PD-1-mediated tumor regression

**Figure 5:**
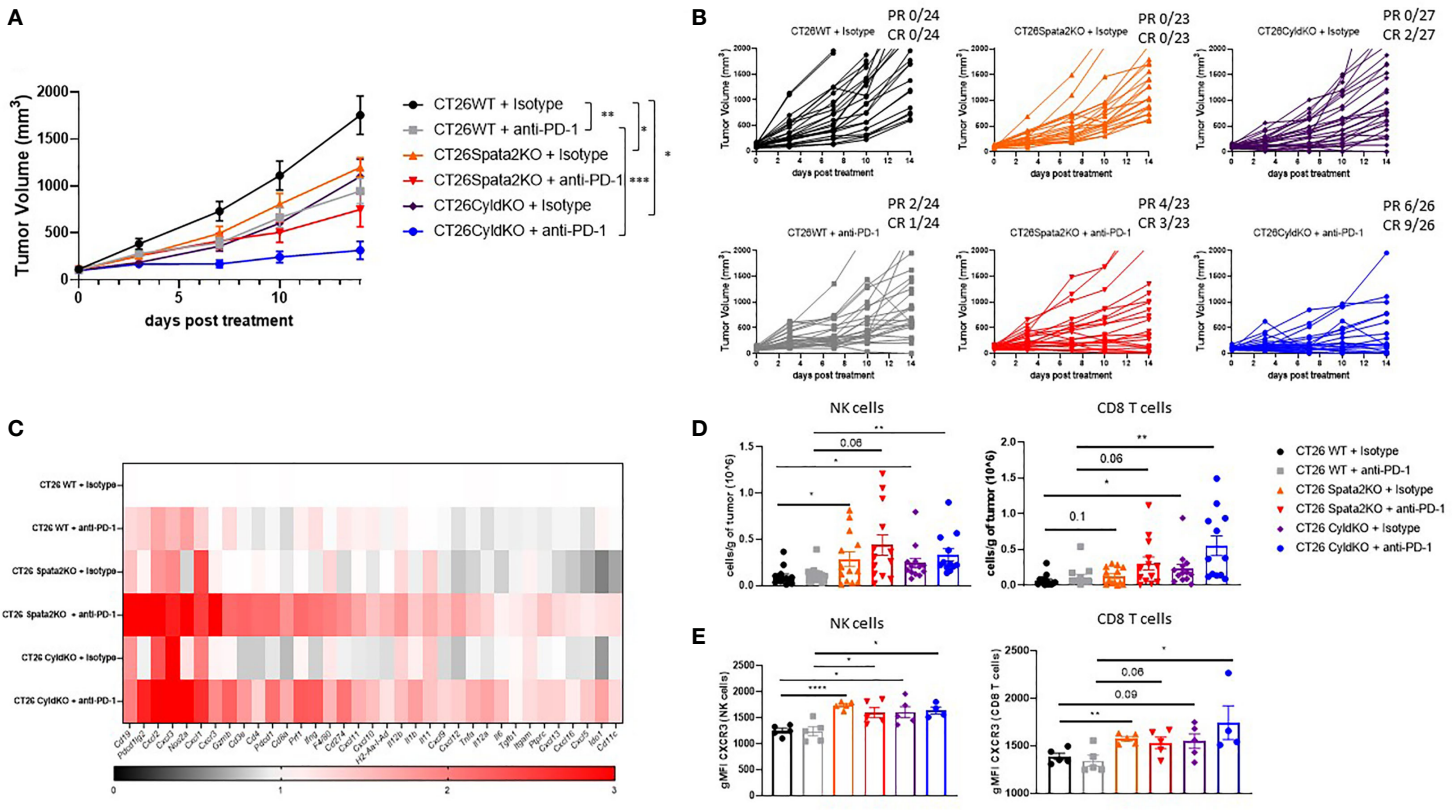
CYLD deficiency enhances anti-PD-1-mediated tumor regression. **(A)** When tumors reached an average size of 100 mm^3^, CT26 tumor-bearing mice were dosed twice weekly with the indicated antibody (at 10 mg/kg) for four doses. **(B)** Individual tumor growth curves. The combined results from 3 independent experiments (n=23-27 mice per group) are shown. **(C)** Gene expression in whole tumors from mice on day 4 after the indicated treatments. Fold-change over isotype control values represented. Results are representative of 2 independent experiments (n=8 mice per group). **(D)** Absolute number of CD8^+^ T cells (left graph) and NK cells (right graph) per gram of tumors was assessed by flow cytometry on day 8 post treatment. Results are representative of 3 independent experiments (n=13 mice per group). **(E)** CXCR3 expression level on CD8^+^ T cells (left graph) and NK cells (right graph) was evaluated by flow cytometry on day 8 post treatment. Results are representative of 1 out of 2 independent experiments (n=5 mice per group). * p<0.05, **p<0.01, *** p<0.001 (Unpaired Student’s *t* test) **(A, D)**. Mean ± SEM.

Finally, we assessed if ablation of SPATA2 and CYLD can ameliorate immune exclusion in CRC tumors by evaluating tumor growth and intratumoral immune infiltration in BALB/c mice implanted with *Spata2*-KO, *Cyld*-KO, or WT CT26 cells. SPATA2 and CYLD deficiency significantly reduced tumor growth in BALB/c mice (**Figures 5A, B**) but not in *Rag2* ^-/-^ BALB/c mice (**Supplementary Figure 3**), suggesting that adaptive immune cells were required for tumor regression. Moreover, SPATA2 and CYLD deficiency did not significantly impact tumor-intrinsic fitness, as evidenced by comparable cell proliferation kinetics *in vitro* between KO and WT cells (**Supplementary Figure 4**). As previously shown (24), anti-PD-1 monotherapy confers partial tumor growth inhibition in mice implanted with CT26 cells. Strikingly, tumor regression was drastically enhanced in *Cyld*-deficient tumor-bearing mice treated with anti-PD-1, and most of the mice achieved a complete (9/26) or partial response (6/26) two weeks post treatment (**Figures 5A, B**). Although anti-PD-1 treatment in *Spata2*-deficient tumor-bearing mice exerted a modest, but not significant, effect on delaying tumor growth, more mice achieved a complete (3/23) or partial response (4/23) compared to anti-PD-1 treatment in WT tumor-bearing mice (1/24 CR; 2/24 PR) two weeks post treatment (**Figures 5A, B**).

Gene expression profiling of tumors from mice showed a profound upregulation of pro-inflammatory cytokines such as *Ifng*, *Prf1*, *Gzmb*, chemokines like *Cxcl9, Cxcl10* and *Cxcl11*, and T cell markers such as *Cd3e*, *Cd8a*, *Cd4*, in SPATA2-deficient and CYLD-deficient tumor-bearing mice treated with anti-PD-1 compared to WT anti-PD-1-treated mice (**Figure 5C**). Increased immune cell infiltration in these tumors was validated by flow cytometry (**Figure 5D**, **Supplementary Figure 5**). In particular, CD8^+^ T cells and natural killer (NK) cells in *Cyld*-KO tumors were significantly more abundant relative to those in WT tumors in control mice, and this difference in cell numbers was amplified with PD-1 blockade, in agreement with the high efficacy of anti-PD-1 treatment in *Cyld-*KO tumors. In addition, an upregulation of CXCR3 expression was observed on tumor-infiltrating CD8^+^ T cells and NK cells in *Spata2*-KO and *Cyld*-KO tumors relative to WT tumors (**Figure 5E**), suggesting a potentiated CXCL9/10/11-CXCR3 signaling axis in recruiting the aforementioned cell subsets into the tumor. These data support the hypothesis that SPATA2 and CYLD promote tumor progression *in vivo*, in part by restraining cytotoxic immune cell infiltration into the TME.

## Discussion

MSS CRC is refractory to current immunotherapies, including anti-PD-1 (33). In contrast to MSI-H CRC, MSS CRC is considered a “cold” tumor type with poor cytotoxic immune cell infiltration (34). The concept of turning cold tumors into hot ones has emerged decades ago. The term “hot tumors” refers to those tumors which have large proportion of tumor-infiltration cytotoxic immune cells and is often associated with a better response to immune checkpoint blockade in the clinic (35). Multiple approaches have been investigated including CXCL9, CXCL10 or CXCL11 overexpression to increase the infiltration of anti-tumoral cells such as cytotoxic CD8 T cells and NK cells (36). In this study, we searched for novel regulators of CXCL9/10/11 expression and T-cell-inflamed GEP in CRC samples using the TCGA gene expression data and identified SPATA2 and CYLD as inhibitors of the aforementioned chemokines in tumor cells (**Figure 1**). Although SPATA2 and especially CYLD have been mainly studied in the context of TNF-α signaling (26–29), little is known about the regulation of IFN-γ signaling by these two proteins. Here we showed a redundant role of SPATA2 and CYLD in the regulation of STAT1 activation and IFN-γ-induced genes (**Figure 2**) in tumor cells.

Previous reports suggested that CYLD impairs STAT1 activation in bone marrow-derived macrophages (37) or in thymic epithelial cells (38) but the mechanism underlying this regulation is still unclear. CYLD is a K63 deubiquitinase activated by SPATA2 that regulates the ubiquitination of proteins involved in TNF-α signaling such as RIPK1, TNFR1, and TRADD (39). Counter-intuitively, we found that STAT1 is polyubiquitinated in WT but not *Spata2*-KO or *Cyld*-KO cells (**Figure 4**), suggesting either a context-dependent protein ubiquitination by CYLD and/or the upregulation/activation of a ubiquitinase, for which STAT1 is a substrate, by CYLD or SPATA2. Recently, Zuo Y. and colleagues showed that HOIP and OTULIN, two protein partners of SPATA2 and CYLD, reciprocally regulate STAT1 linear ubiquitination (40), suggesting context-dependent regulation of protein ubiquitination by SPATA2 and CYLD. Internal data further suggest that SPATA2 and CYLD trigger K63- but not K48-specific ubiquitination of STAT1 (data not shown). Although K48 ubiquitination triggers proteasomal degradation, some reports suggest that K63 ubiquitination regulates both proteasome-independent signaling pathways and proteasome-mediated protein degradation (41, 42). Thus, reduced K63-ubiquitination of STAT1 in the absence of SPATA2 or CYLD might result in reduced proteasomal degradation of STAT1.

Although SPATA2 has recently emerged as an activator of CYLD, suggesting redundancy between the two proteins, several studies also highlighted distinct functions of these two proteins (27). For instance, the ubiquitination of RIPK1 and TNFR1 was reduced in the absence of SPATA2 (26, 29). In contrast, an increased ubiquitination of RIPK1 and TNFR1 was observed in CYLD-deficient cells (39). Even more strikingly, while CYLD-deficient mice spontaneously developed lung and colonic inflammation associated with an increase of T cell activation (32), SPATA2-deficient mice are reportedly healthy (25), suggesting a differential role for the two proteins in immune cell regulation. More in-depth studies are required to fully understand the redundant versus non-redundant roles of SPATA2 and CYLD across various cell types and their implications in human disease. The development of specific inhibitors will help address this question.

Numerous splicing variants in CYLD have been identified. Interestingly, full-length CYLD and a CYLD variant lacking exons 7 and 8 (CYLD^ex7/8^) appear to mediate opposing functions, with full-length CYLD antagonizing NF-κB and MAPK signaling and CYLD^ex7/8^ exerting the opposite effects (43–45). Moreover, CYLD variants might have differential patterns of expression across cell types and tissue contexts. For instance, CYLD^ex7/8^ expression and activity has been primarily described in immune cells (43–46), and Tang and colleagues reported increased CYLD splicing in the T cells of Crohn’s Disease patients (44). Whether CYLD splicing plays a role in CRC remains to be determined. In this study, exon 2 of CYLD was targeted using guide RNAs, leading to a complete ablation of protein production and the absence of any of known spliced variants. The effects of different CYLD splice variants and the ratio of these variants on STAT1 accrual and signaling in CRC tumor cells merit future investigation.

In summary, we identified SPATA2 and CYLD as novel regulators of T/NK cell-attracting chemokines CXCL9, CXCL10, CXCL11 in tumor cells. Mechanistically, we found that SPATA2 and CYLD prevent STAT1 accumulation and activation *in vitro*. Genetic deletion of Spata2 and CYLD enhances infiltration of anti-tumoral CXCR3 T/NK cells and anti-PD-1-mediated tumor regression *in vivo.* Our results raise the intriguing possibility of therapeutically targeting the SPATA2-CYLD axis to improve responses of colorectal cancer to PD-(L)1 blockade and other immunotherapies. As CYLD is expressed in multiple cell types beyond tumor cells, including T and B cells, with potentially pleiotropic functions across cell types, it would be important to improve CYLD-targeting specificity (47) with tumor-targeting strategies such as antibody drug conjugates to reduce on-target, off-tumor toxicity to achieve both therapeutic efficacy and safety.

## Data availability statement

The original contributions presented in the study are included in the article/**Supplementary Material**. Further inquiries can be directed to the corresponding author.

## Ethics statement

The animal study was reviewed and approved by All animal procedures were approved by the South San Francisco Institutional Animal Care and Use Committee in accordance with guidelines of the Association for Assessment and Accreditation of Laboratory Animal Care.

## Author contributions

TT, MW, AC, DB designed the studies. TT, DB, YZ, AO, CM, SJ performed *in vitro, in silico, ex vivo* and *in vivo* experiments. SS provided helpful discussion. DB, TT, AC wrote the manuscript. AC and DB supervised the project. All authors contributed to the article and approved the submitted version.

## Funding

The study was sponsored by Merck Sharp & Dohme Corp., a subsidiary of Merck & Co., Inc., Kenilworth, NJ, USA.

## Acknowledgments

We would like to acknowledge Merck Sharp & Dohme Corp., a subsidiary of Merck & Co., Inc., Kenilworth, NJ, employee: Dewan Hossain for reviewing the manuscript, Galya Vassileva for coordinating CRISPR KO cell lines generation and Chloe Vaughn and Galina Kosikova for their technical support.

## Conflict of interest

The study was sponsored by Merck Sharp & Dohme Corp., a subsidiary of Merck & Co., Inc., Kenilworth, NJ, USA and that the authors are Merck Sharp & Dohme Corp., a subsidiary of Merck & Co., Inc., Kenilworth, NJ, USA employees.

## Publisher’s note

All claims expressed in this article are solely those of the authors and do not necessarily represent those of their affiliated organizations, or those of the publisher, the editors and the reviewers. Any product that may be evaluated in this article, or claim that may be made by its manufacturer, is not guaranteed or endorsed by the publisher.

## Supplementary material

Supplementary Figure 1 Production of CXCL9, CXCL10, and CXCL11 from human CRC cell lines upon knockdown of nominated T-cell-exclusionary targets and *CYLD*. LS411N and SW480 were treated with siRNAs against the indicated targets with or without IFN-γ stimulation as described in . **(A)** Levels of indicated chemokines upon knockdown of the indicated targets. **(B)** Fold-change of indicated chemokines upon knockdown of indicated targets relative to control-siRNA-treated cells. All cells were treated with IFN-γ. Bars are ordered as per , right panel. Mean + SEM. * p<0.05, ** p<0.01, *** p<0.001. Student’s *t* test **(A)**, One-sample Student’s *t* test **(B)**.

Supplementary Figure 2 SPATA2 and CYLD do not regulate JAK1 and IκBa expression in CT26 cells. JAK1 and IκBα expression in WT, *Spata2*-KO, and *Cyld-*KO CT26 cells was measured by Western blot. Results are representative of 3 independent experiments. ns: not significant (One way-ANOVA). Mean ± SEM.

Supplementary Figure 3 Adaptive immune cells are required for SPATA2 and CYLD-mediated tumor growth. *Rag2* ^-/-^ BALB/c mice were implanted subcutaneously with CT26 cells as indicated. Tumor volumes were measured twice weekly. Results are representative of 1 experiment (n=10 mice per group). Mean ± SEM.

Supplementary Figure 4 Deficiency of SPATA2 or CYLD does not impair tumor cell proliferation *in vitro.* Cell proliferation was assessed *in vitro.* Results are representative of 3 independent experiments. Mean ± SEM.

Supplementary Figure 5 Immunophenotyping of CT26 tumors. **(A)** Gating strategy to identify immune cell infiltration in tumors. **(B)** Absolute number of CD45+ immune cells (top left graph), conventional CD4 T cells (top right graph), gMDSCs (bottom left graph) and mMDSCs (bottom right graph) per gram of tumors was assessed by flow cytometry at day 8 post treatment. Results are representative of 3 independent experiments (n=13 mice per group). ns: not significant, * p<0.05 (Unpaired Student’s *t* test). Mean ± SEM.

Supplementary Table 1 Correlation coefficients and *p* values of all genes with CXCL9/10/11 and GEP scores in TCGA. Expression levels in CCLE CRC cell lines and genes selected for downstream validation are also indicated.
