# Supplementary figures and images for: SPATA2 and CYLD inhibit T cell infiltration into colorectal cancer via regulation of IFN-γ/STAT1 axis
Source: Front Oncol. 2022 Dec 2;12:1016307. doi: 10.3389/fonc.2022.1016307 (PMC9756846; doi:10.3389/fonc.2022.1016307)

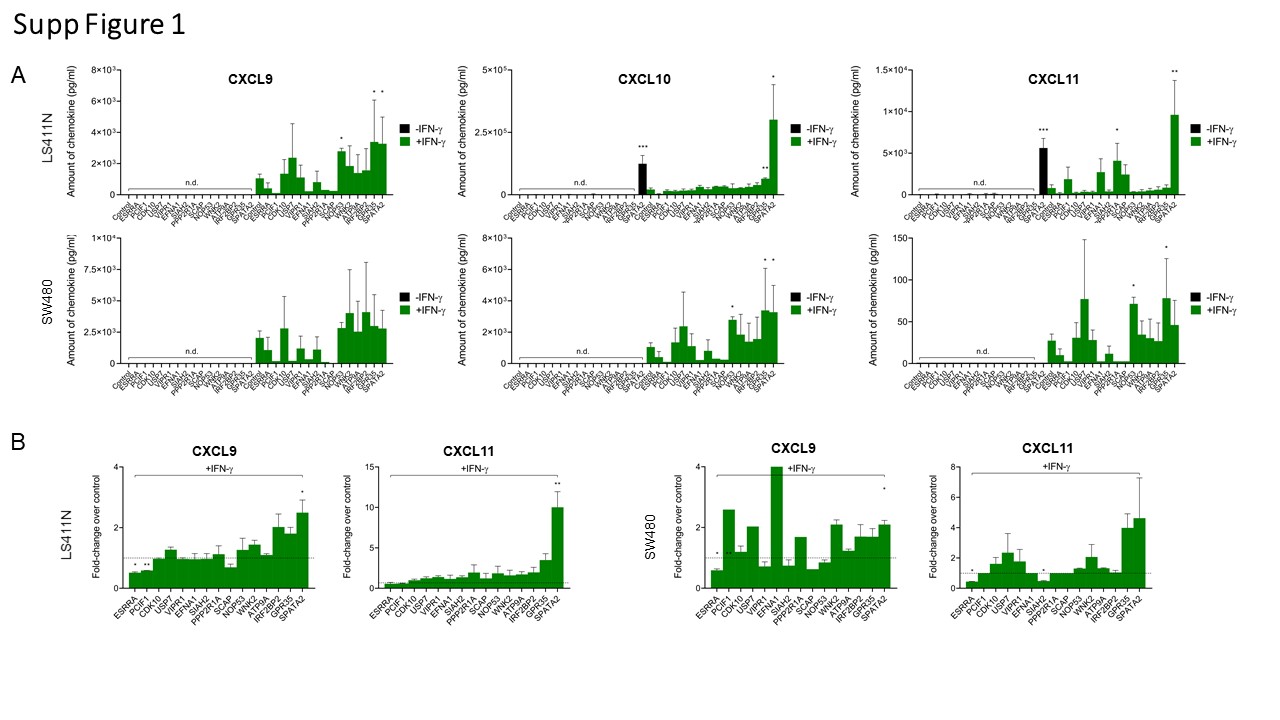

Supplement: Supplementary Figure 1 — Production of CXCL9, CXCL10, and CXCL11 from human CRC cell lines upon knockdown of nominated T-cell-exclusionary targets and CYLD. LS411N and SW480 were treated with siRNAs against the indicated targets with or without IFN-γ stimulation as described in . (A) Levels of indicated chemokines upon knockdown of the indicated targets. (B) Fold-change of indicated chemokines upon knockdown of indicated targets relative to control-siRNA-treated cells. All cells were treated with IFN-γ. Bars are ordered as per , right panel. Mean + SEM. * p<0.05, ** p<0.01, *** p<0.001. Student’s t test (A), One-sample Student’s t test (B). [file Image_1.jpg]

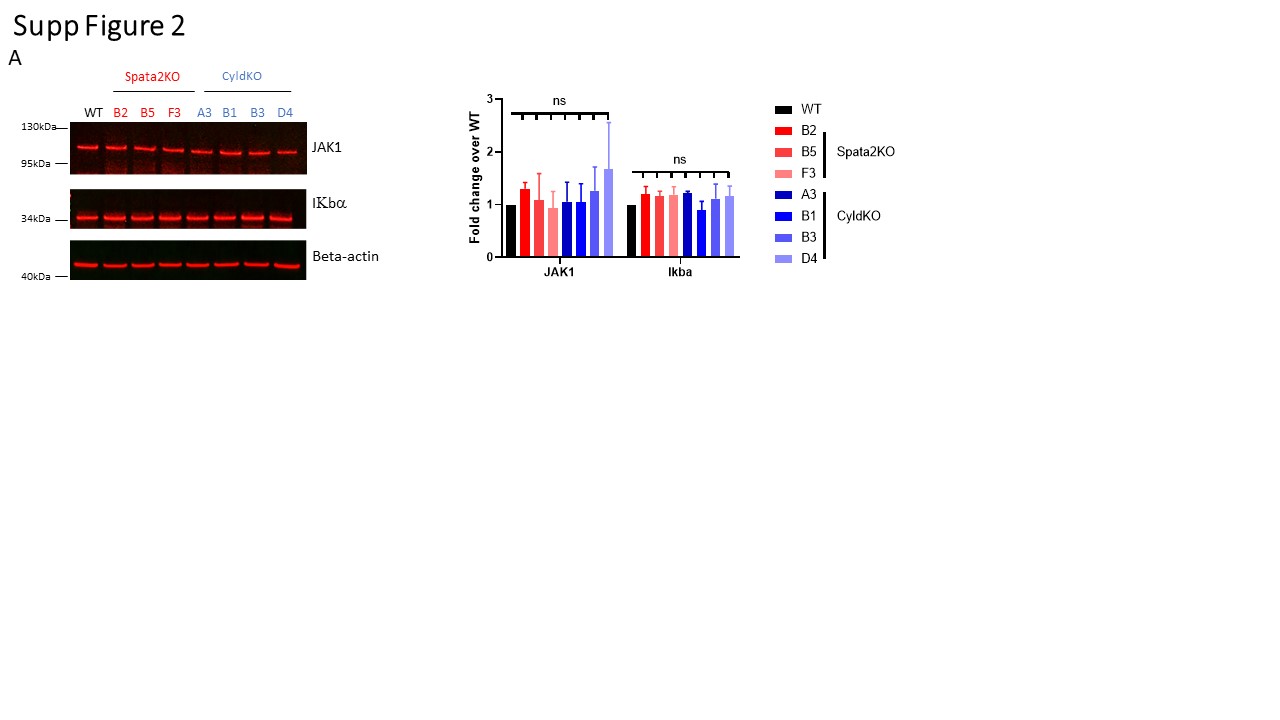

Supplement: Supplementary Figure 2 — SPATA2 and CYLD do not regulate JAK1 and IκBa expression in CT26 cells. JAK1 and IκBα expression in WT, Spata2-KO, and Cyld-KO CT26 cells was measured by Western blot. Results are representative of 3 independent experiments. ns: not significant (One way-ANOVA). Mean ± SEM. [file Image_2.jpeg]

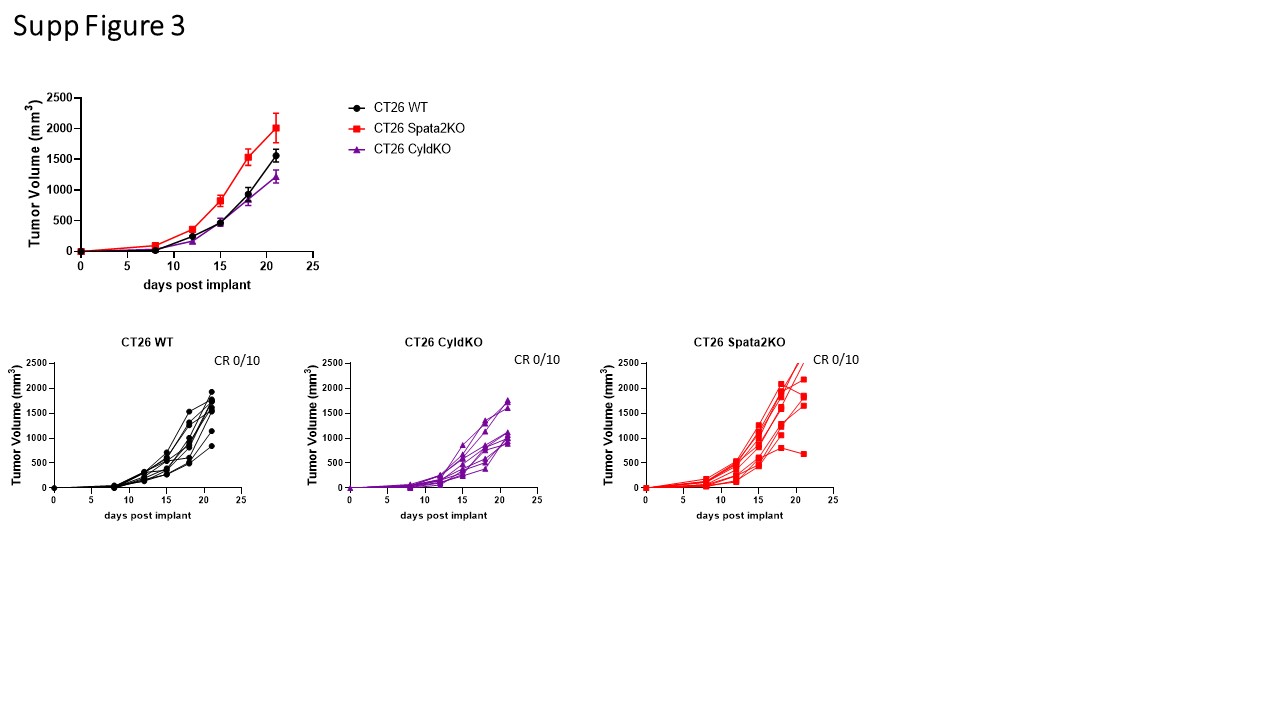

Supplement: Supplementary Figure 3 — Adaptive immune cells are required for SPATA2 and CYLD-mediated tumor growth. Rag2 -/- BALB/c mice were implanted subcutaneously with CT26 cells as indicated. Tumor volumes were measured twice weekly. Results are representative of 1 experiment (n=10 mice per group). Mean ± SEM. [file Image_3.jpeg]

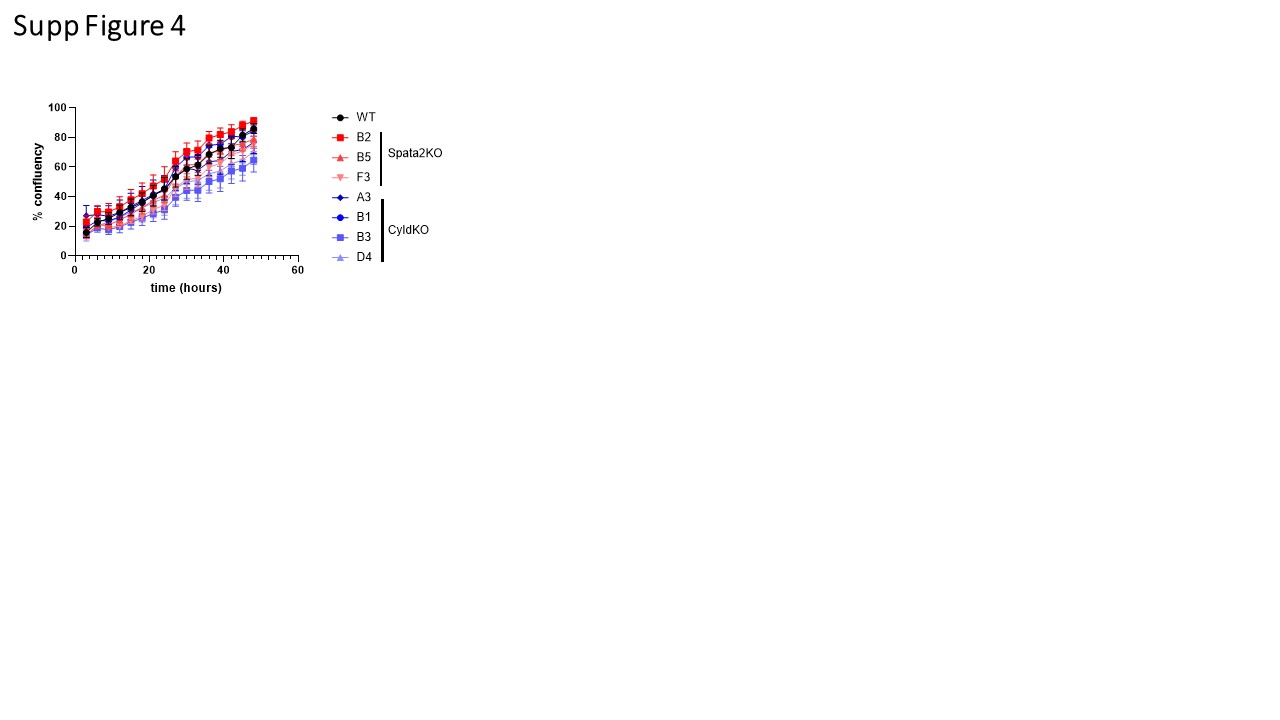

Supplement: Supplementary Figure 4 — Deficiency of SPATA2 or CYLD does not impair tumor cell proliferation in vitro. Cell proliferation was assessed in vitro. Results are representative of 3 independent experiments. Mean ± SEM. [file Image_4.jpeg]

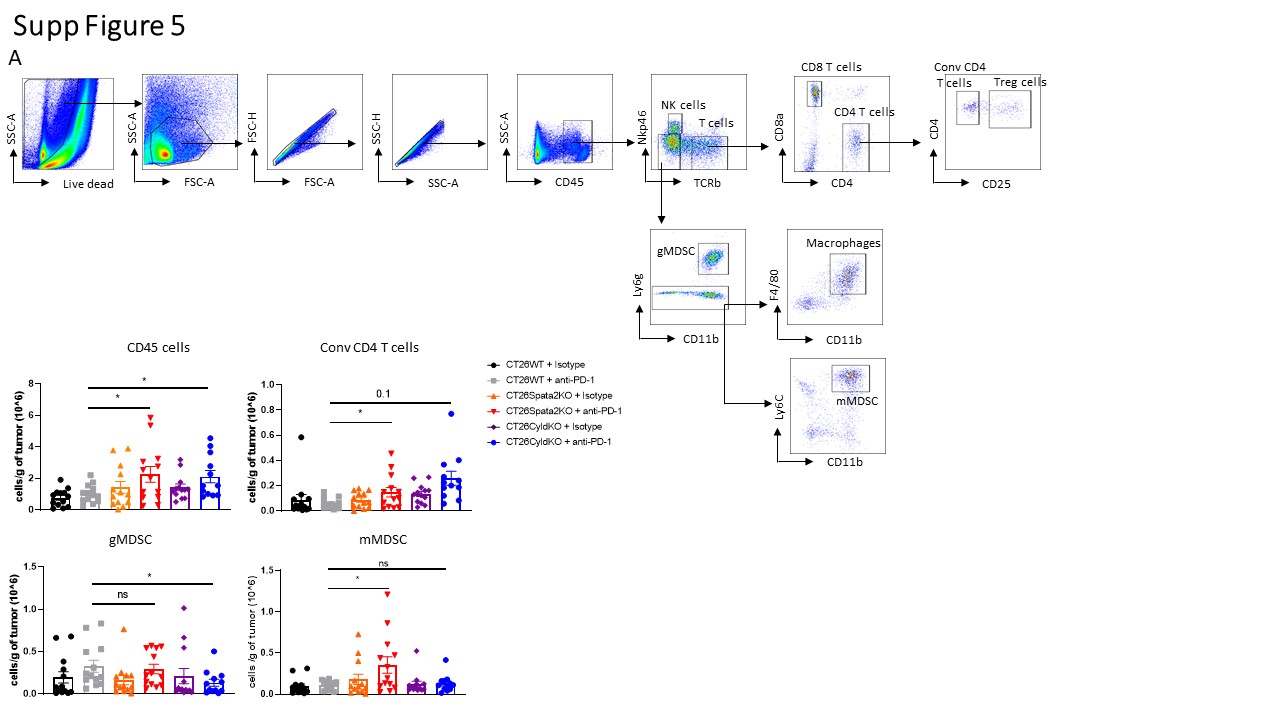

Supplement: Supplementary Figure 5 — Immunophenotyping of CT26 tumors. (A) Gating strategy to identify immune cell infiltration in tumors. (B) Absolute number of CD45+ immune cells (top left graph), conventional CD4 T cells (top right graph), gMDSCs (bottom left graph) and mMDSCs (bottom right graph) per gram of tumors was assessed by flow cytometry at day 8 post treatment. Results are representative of 3 independent experiments (n=13 mice per group). ns: not significant, * p<0.05 (Unpaired Student’s t test). Mean ± SEM. [file Image_5.jpeg]
